# Extractive Clinical Question-Answering With Multianswer and Multifocus Questions: Data Set Development and Evaluation Study

**DOI:** 10.2196/41818

**Published:** 2023-06-20

**Authors:** Sungrim Moon, Huan He, Heling Jia, Hongfang Liu, Jungwei Wilfred Fan

**Affiliations:** 1 Department of Artificial Intelligence & Informatics Mayo Clinic Rochester, MN United States

**Keywords:** question-answering, information extraction, dataset, data set, artificial intelligence, natural language processing

## Abstract

**Background:**

Extractive question-answering (EQA) is a useful natural language processing (NLP) application for answering patient-specific questions by locating answers in their clinical notes. Realistic clinical EQA can yield multiple answers to a single question and multiple focus points in 1 question, which are lacking in existing data sets for the development of artificial intelligence solutions.

**Objective:**

This study aimed to create a data set for developing and evaluating clinical EQA systems that can handle natural multianswer and multifocus questions.

**Methods:**

We leveraged the annotated relations from the 2018 National NLP Clinical Challenges corpus to generate an EQA data set. Specifically, the 1-to-N, M-to-1, and M-to-N drug-reason relations were included to form the multianswer and multifocus question-answering entries, which represent more complex and natural challenges in addition to the basic 1-drug-1-reason cases. A baseline solution was developed and tested on the data set.

**Results:**

The derived RxWhyQA data set contains 96,939 QA entries. Among the answerable questions, 25% of them require multiple answers, and 2% of them ask about multiple drugs within 1 question. Frequent cues were observed around the answers in the text, and 90% of the *drug* and *reason* terms occurred within the same or an adjacent sentence. The baseline EQA solution achieved a best *F*_1_-score of 0.72 on the entire data set, and on specific subsets, it was 0.93 for the unanswerable questions, 0.48 for single-drug questions versus 0.60 for multidrug questions, and 0.54 for the single-answer questions versus 0.43 for multianswer questions.

**Conclusions:**

The RxWhyQA data set can be used to train and evaluate systems that need to handle multianswer and multifocus questions. Specifically, multianswer EQA appears to be challenging and therefore warrants more investment in research. We created and shared a clinical EQA data set with multianswer and multifocus questions that would channel future research efforts toward more realistic scenarios.

## Introduction

### Background

The thought process involved in clinical reasoning and decision-making can be naturally framed into a series of questions and answers [1,2]. Achieving human-like question-answering (QA) capability is highly regarded in artificial intelligence (AI). Medical QA research has garnered terrific momentum over the past decade, and a new generation of AI scientists is undergoing a state-of-the-art update at a daunting pace almost every month (if not every week). One of the very sought-after applications is to find the answer within a given document, or extractive QA (EQA), which enables patient-specific QA based on the information provided in the clinical text [3]. As an essential component in most AI engineering undertakings, EQA training data determine not only the likelihood of success in terms of annotation quality but also the fidelity of representing the target scenario.

Along with other issues observed in existing medical EQA corpora [4], the mainstream annotation approach knowingly simplifies the task into a “one answer per document” scheme. Although the simplification makes development and evaluation easier for promoting initial growth of the field, it is unrealistic because EQA can naturally have multiple qualified answers (or answer components) within 1 document, and often all of them must be captured to sufficiently answer a question [5]. Moreover, a question can naturally involve multiple focus points such as “Why A, B, and C…” rather than requiring the user to ask 1 question for each point. To address this gap, we created an EQA data set that involves realistic, multianswer and multifocus cases by converting the concept-relation annotations from an existing clinical natural language processing (NLP) challenge data set. Our generated RxWhyQA data set includes a total of 96,939 QA entries, where 25% of the answerable questions require the identification of multiple answers and 2% of them ask about multiple drugs within 1 question. We also developed a baseline solution for multianswer QA and tested it on the RxWhyQA.

The novelty of this study is reframing the original relation identification task into an EQA task, which simplifies the conventional 2-step approach of named entity recognition and relation classification into 1-step information extraction guided by natural language questions. Our primary contribution is the RxWhyQA as a resource that offers realistic constructs to facilitate NLP research in this underexplored area. To our knowledge, there has not been any EQA data set that contains multianswer and multifocus questions based on clinical notes.

### Related Work

QA is a versatile task that can subsume diverse NLP tasks when properly represented [6]. More than a decade of research has focused on the EQA task in NLP [7]. As the name implies, EQA can be viewed as question-guided information extraction from a given text. Unlike conventional approaches that require the identification of the involved entities as one task followed by determination of the target relation between the entities as the other task, EQA consolidates these steps into a smooth one-shot task where the user asks a natural language question for the system to understand the focus point, identify relevant cues in the text, and locate the answer that satisfies the relation of interest. Although EQA demands higher machine intelligence, it is efficient in terms of the data schema for modeling and the human-computer interaction for users.

The Stanford Question Answering Dataset (SQuAD) [8] established a widely adopted framework for EQA, and in the later version (version 2.0) [9], the task also requires a system to refrain from answering when no suitable answer is present in the text. In the clinical domain, corpora have been developed for EQA based on electronic health records (EHRs). In the study by Raghavan et al [10], medical students were presented with structured and unstructured EHR information about each patient to generate realistic questions for a hypothetical office encounter. Using the BioASQ data set based on biomedical literature, Yoon et al [5] proposed a sequence tagging approach to handling multianswer EQA. In the consumer health domain, Zhu et al [11] developed a Multiple Answer Spans Healthcare Question Answering (ie, MASH-QA) data set specifically involving multiple answers of nonconsecutive spans in the target text. As a non-English example, Ju et al [12] developed a Conditional Multiple-span Chinese Question Answering data set from a web-based QA forum. Pampari et al [13] developed the emrQA, a large clinical EQA corpus generated through template-based semantic extraction from the Informatics for Integrating Biology & the Bedside NLP challenge data sets. We took a similar approach as the emrQA but additionally included multianswer and multifocus questions that better reflect natural clinical EQA scenarios.

## Methods

### Generating the QA Annotations From a Relation Identification Challenge

Our source data were based on the annotations originally created for the National NLP Clinical Challenges (n2c2) corpus of 2018, which aimed to identify adverse drug events by extracting various drug-related concepts and classifying their relations in the clinical text [14]. Their final gold standard included 83,869 concepts and 59,810 relations in 505 discharge summaries. In this study, we focused on generating QA pairs from the subset of drug and reason concepts (ie, mainly about the prescribing justification) and the relations between the concepts. Each relation consisted of 2 arguments: a drug concept and a reason concept, as in an example pair such as *drug-reason* (morphine-pain). Accordingly, a question around the drug concept could be derived, such as “Why was morphine prescribed to the patient?” and the reason concept “pain” would be designated as the answer. In the n2c2 corpus, each pair of drug and reason concepts had their text mentions annotated in the corresponding clinical document. The properties make for a good EQA data set where the system is expected to consider the actual contexts surrounding the drug and reason rather than performing a simple lookup. This is especially important for extracting off-label uses because a standard indication knowledge base would not cover those exceptions documented in real-world clinical text.

From the n2c2 annotations on each clinical document, we leveraged several relation types between the drug and reason concepts: 1 drug 0 reason, 1 drug 1 reason, 1 drug N reasons, N drugs 1 reason, or M drugs N reasons. The most straightforward were the 1-drug-1-reason relations (eg, the morphine-pain relation mentioned above), each translated into a 1-to-1 QA entry. The 1-drug-0-reason relations apparently corresponded to the 1-to-0 (unanswerable) QA entries. We preserved the 1-drug-N-reasons relations directly as 1-to-N QAs that require locating multiple answers in the text. For the N-drugs-1-reason and M-drugs-N-reasons relations, we preserved the original multidrug challenge in questions such as, “Why were amlodipine, metoprolol, and isosorbide prescribed to the patient?” The M-drugs-N-reasons relations would also derive multianswer entries such as those derived from the 1-drug-N-reasons relations. In addition to the generated QA entries, we also supplemented paraphrastic questions [15] that may enhance the generalizability of the trained systems.

### Quantitative and Qualitative Analysis of the Derived QA Annotations

Along with descriptive statistics of the QA entries and the number of answers per question, we computed the frequencies of the specific drug and reason concept terms (after applying lexical normalization such as lowercase) among the QA entries. The frequencies were meant to offer an intuitive estimate of the abundance of train/test data available for each specific concept or concept pair. We then randomly sampled 100 QA entries for manual review: 50 from those with a single answer and 50 from those with multiple answers. The common patterns informative to QA inference were summarized, offering evidence on what the potential AI solutions could leverage. In addition, we measured the distance (by the number of sentences) between the question and answer concepts. For each specific drug-reason pair, we considered the shortest distance if there were multiple occurrences of either concept. The distance was deemed 0 if the pair occurred within the same sentence. Distance may serve as a surrogate for measuring the challenge to AI systems, where a longer distance implies a more challenging task. In addition, we sampled 100 random drug-reason pairs from each test run (experimental setup described below) to estimate the prevalence of off-label uses in the derived data set. The MEDication-Indication (MEDI) knowledge base (version 2) high-precision subset [16] was first used to screen for on-label uses by exact string match (with normalizing to lowercase), and the remaining drug-reason pairs were reviewed by a domain expert (HJ) to determine off-label uses.

### Development of a Baseline Solution

#### Data Preparation and Model Training

The annotations conform to the SQuAD 2.0 JSON format and can be readily used to train Bidirectional Encoder Representations from Transformers (BERT) [17] for EQA tasks. We randomly partitioned the data set into the train, develop (dev), and test sets by the 5:2:3 ratio, corresponding to 153, 50, and 100 clinical documents, respectively. Random partitioning was carried out 3 times, each executed as a separate run of the experiment for quantifying performance variability. The base language model was ClinicalBERT [18], a domain-customized BERT trained on approximately 2 million clinical documents from the MIMIC-III (version 1.4) database. We fine-tuned ClinicalBERT first on a why-question subset of SQuAD 2.0, followed by fine-tuning on the train set. Training parameters used in the ClinicalBERT fine-tuning were batch_train_size=32, max_seq_length=128, doc_stride=64, learning_rate=3e-5, and epochs=5. The dev set was then used to learn the threshold for determining when the ClinicalBERT model should refrain from providing any answer.

#### Incremental Masking to Generate Multiple Answers

To force the fine-tuned ClinicalBERT model to continue seeking other suitable answers in each clinical document, we implemented the following process on the test set as a heuristic baseline:

1. Let the EQA model complete its usual single-answer extraction and record the string of the top answer. No further action is needed if the model refrains from answering.
2. Perform a case-insensitive string search using the top answer (from step 1 above) throughout the clinical note from where it was extracted and replace every occurrence into a dummy underscore “______” string of identical length. This literally generates a new version of the text by masking the original top answer in each question.
3. Run the same EQA model for another round on the entire masked test set again to determine whether the model could identify additional answers elsewhere or started to refrain from answering.

**Figure 1:**
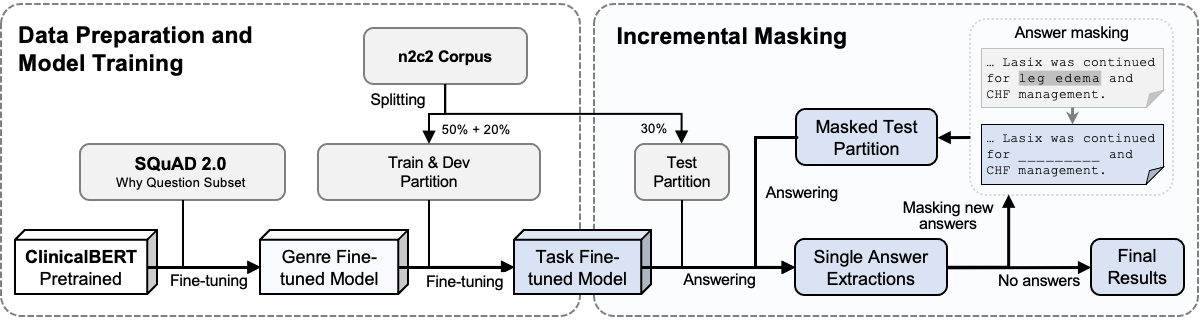
A flowchart of our heuristic approach to constructing a single-answer extractive question-answering model generates multiple answers by incremental masking. The main steps go from left to right. The upper-right “Answer-masking” box illustrates an example of masking where the model’s answer “leg edema” is replaced with a dummy underscore to force the model to look for viable alternative answers elsewhere in the text. BERT: Bidirectional Encoder Representations from Transformers; dev: develop; n2c2: National NLP Clinical Challenges; NLP: natural language processing; SQuAD: Stanford Question Answering Dataset.

The 3 abovementioned steps were repeated until the model did not generate any new answers on the entire test set. Together, model training and the heuristic multianswer generation process are summarized in Figure 1.

### Evaluation of the Baseline Solution

After the first round of masking, we began to have more than 1 answer generated by the model for some of the questions. Accordingly, the evaluation program (specifically for the overlap mode) was adapted to accommodate such M-to-N answer comparisons in determining the token-wise proportional match. When anchoring on each gold-standard answer, we selected the model answer with the most overlapping tokens as the best answer in setting the weighted true positive (TP) and false negative (FN); the weighted false positive (FP) was set vice versa by anchoring on each model answer—see equations 1-4 for definitions. On top of these weighted matches between gold-standard and model answers in each question, we tallied them over each entire test set to compute the solution’s precision, recall, and *F*_1_-score, followed by qualitative error analysis.



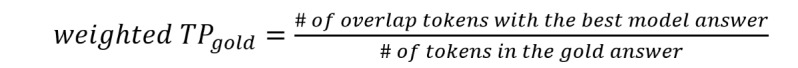





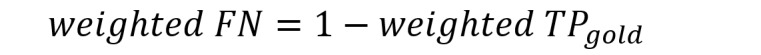





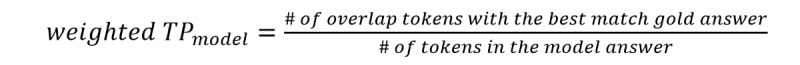





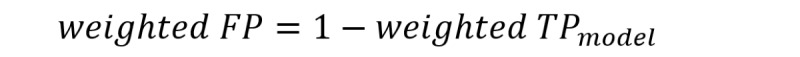



## Results

### Descriptive Statistics of the Derived RxWhyQA Data Set

**Figure 2:**
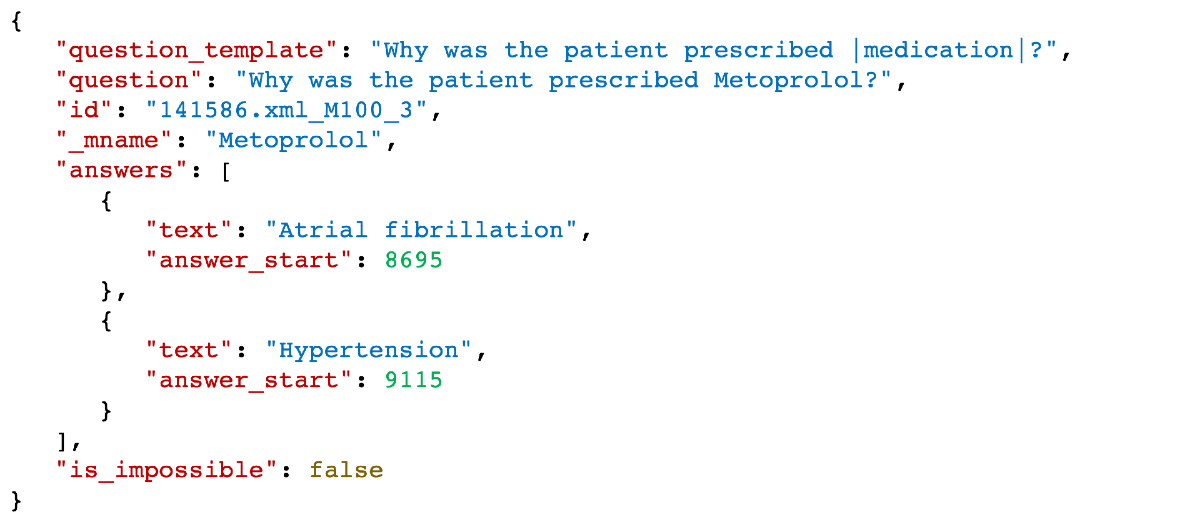
A multianswer entry in the generated RxWhyQA data set. The “id” field is the unique ID for the question-answering entry in the data set. The “_mname” field indicates the medication name; that is, the anchor concept in the question. The “answer_start” is the character offset where the answer term occurs in the clinical document, which is hosted in the “context” field (not shown here). When “is_impossible” is false, the question-answering entry is answerable.

We leveraged a total of 10,489 relations from the n2c2 adverse drug events NLP challenge and derived the data set, consisting of 96,939 QA entries. Table 1 summarizes the 5 major drug-reason relation categories in the n2c2 corpus, the strategies that we implemented to convert them into QA entries, and their resulting frequencies. Table 2 shows the distribution for the number of answers per question: 75% of the questions have a single answer, while 25% of them require multiple answers. Duplicate answer terms located at different positions of the clinical documents were retained. For example, the procedure “CT” might be mentioned at several places in the text and be recorded as the answer to “Why was the patient prescribed contrast?” We included each such identical term and their different offsets as multiple answers so that the EQA solutions may leverage such nuances. The final data set was formatted into a SQuAD-compatible JSON file and shared through the n2c2 community annotations repository [19]. Figure 2 illustrates a multianswer entry in the RxWhyQA data set.

**Table 1:** Categories, examples, and conversion strategies for making the drug-reason relations into the extractive question-answering annotations.

Category in the n2c2^a^ corpus	Example	Conversion strategy	Entries, n
1 *Drug*, no Reason	*Mirtazapine* 15 mg PO QHS^b^ (only the drug is mentioned but no reason is documented)	Make an unanswerable QA^c^ entry	46,278
1 *Drug*, 1 Reason	The patient received *morphine* for pain as needed	Make a 1-to-1 QA entry	28,224^d^
N *Drugs*, 1 Reason	Hypertension: severely elevated blood pressure. Started *amlodipine*, *metoprolol*, and *isosorbide*.	Break into N separate 1-to-1 relations and make each a 1-to-1 QA entry	N/A^e^
1 *Drug*, N Reasons	*Albuterol sulfate* 90 mcg… Puff Inhalation Q4H^f^ for sob or wheeze.	List the N reasons under the answer block to form a 1-to-N QA entry	22,437^g^
M *Drug*, N Reasons	Left frontoparietal stroke - maintained on *ASA*^h^ and *plavix ….* Hx of CVA^i^*:* restarted *ASA/Plavix* per the GI^j^ team's recommendation*.*	List the N reasons under answer block to form an M-to-N QA entry	N/A

^a^n2c2: National NLP (natural language processing) Clinical Challenges.

^b^PO QHS: one pill to be taken orally at bedtime.

^c^QA: question-answering.

^d^28,224 entries in total for the 1-drug-1-reason and N-drugs-1-reason categories together in the corpus.

^e^N/A: not applicable.

^f^Q4H: every 4 hours.

^g^22,437 entries in total for the 1-drug-N-reasons and M-drug-N-reasons categories in together in the corpus.

^h^ASA: acetylsalicylic acid (aspirin).

^i^Hx of CVA: history of cerebrovascular accident.

^j^GI: gastrointestinal.

**Table 2:** Unique answers among answerable questions.

Frequency	Unique answers, n (%)
1	28,224 (75)
2	6804 (18)
3	1530 (4)
≥4	954 (3)

### Content Analysis of the RxWhyQA Data Set

The 5 most frequently asked drug terms (with noting the number of QA entries) in the answerable questions (frequencies) were the following: coumadin (1278), vancomycin (1170), lasix (963), acetaminophen (801), and antibiotics (783). Without any overlap, the 5 most frequent drug terms in the unanswerable questions were the following: docusate sodium (648), metoprolol tartrate (504), aspirin (468), pantoprazole (450), and penicillins (414). Among the answerable QA entries, the 5 most frequently seen pairs were the following: acetaminophen-pain (504), senna-constipation (369), oxycodone-pain (261), coumadin-afib (252), and acetaminophen-fever (234). As a potential surrogate measure of task difficulty, Table 3 shows the distribution for the number of sentences between the question anchor and answer term in each answerable QA entry. The majority (n=32,409, 72%) of the drug and reason terms occur within the same sentence, and the portion increases to 90% (72%+18%) when adding those with the drug and reason occurring in an adjacent sentence (ie, distance=1). In the extreme case, the drug and reason terms are 16 sentences apart from each other. Table 4 summarizes the commonly observed contexts from manually reviewing 100 random samples of the answerable QA entries. There were 7, 10, and 3 off-label uses, respectively, in each of the random 100 drug-reason pairs reviewed by the domain expert, making the estimate of off-label uses average at 6.7% in the RxWhyQA data set. The detailed off-label review results are available in Multimedia Appendix 1.

**Table 3:** Distribution for the distance between question and answer terms (0=the question and answer terms occur in the same sentence).

Distance (be sentence) between the question and answer items	QA^a^ entries, n
0	32,409
1	8154
2	2646
3	1188
4	405
5	153
6	81
7	72
8	27
9	0
10	0
11	0
12	9
13	9
14	0
15	0
16	9

^a^QA: question-answering.

**Table 4:** Common patterns (observed >10 times) between the question and the answer terms in 100 random question-answering entries. Each reason or drug represents where a question or answer anchor term occurs in the pattern. The shorthands are used as follows: ellipsis stands for 0 to multiple words, parentheses denote scoping, square brackets with pipes indicate a boolean OR set, and a question mark denotes a binary quantifier for presence or absence.

Pattern	Frequency
Reason … (being)? [received|started|restarted|required|maintained|continued?] (on)? *Drug*	25
*Drug* … [prn|PRN|(as needed for)?] Reason	18
*Drug* … (was)? [attempted|given|dosing|taking] for (any)? [possible|likely|presumed]? Reason	14
Reason … (was)? [managed|treated|improved|recommended|downtrended|resolved|reversed|needed] with *Drug*	13

### F1-Score of the Baseline EQA Solution

**Figure 3:**
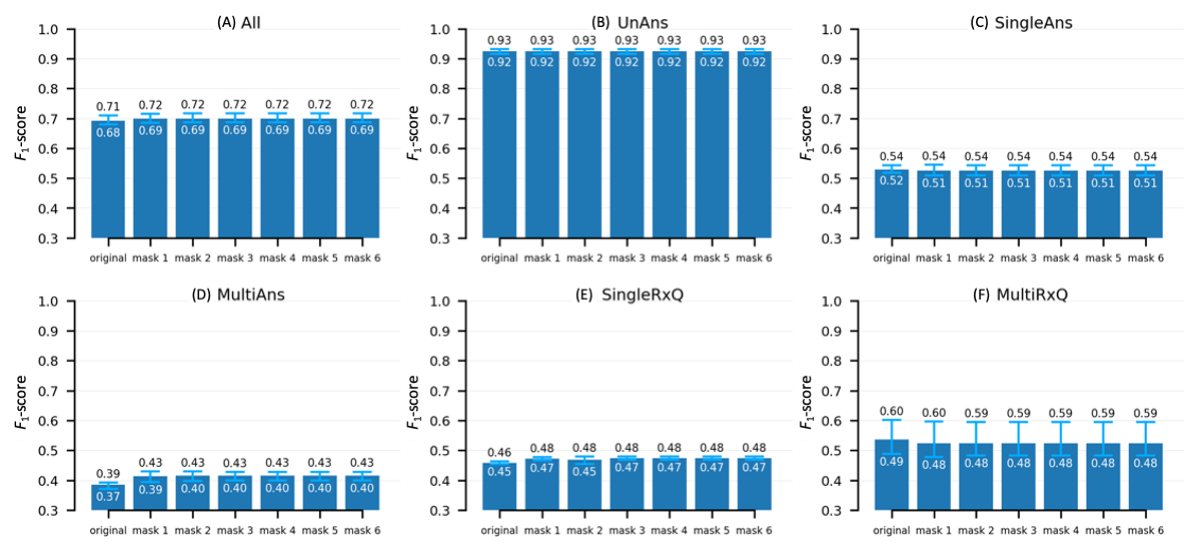
*F*_1_-scores of the fine-tuned Bidirectional Encoder Representations from Transformers extractive question-answering model across the incremental masking rounds. Each bar represents the average *F*_1_-score based on 3 experimental runs, with the minimum and maximum range marked (light blue). (A) The full set, (B) unanswerable questions, (C) questions with exactly 1 answer, (D) questions with multiple answers, (E) questions asking about a single drug, and (F) questions asking about multiple drugs.

The performance in determining the *F*_1_-score across 3 experimental runs is summarized in Figure 3, where the subfigures represent different slices. Specifically, the underlying set relations are the following: the full set (Figure 3A) minus the unanswerable questions (Figure 3B) yields the answerable questions, which can be represented by either single-answer questions (Figure 3C) plus multianswer questions (Figure 3D) if sliced per the number of answers or by questions asking about a single drug (Figure 3E) plus questions asking about multiple drugs (Figure 3F) if sliced per the number of drugs asked in the question. Each bar represents the average *F*_1_-score across the runs and with the range marked for each incremental masking step. As seen in Figure 3A, the overall *F*_1_-score increased immediately after applying the first round of answer masking (from “original” to “mask 1”, *P*<.05), which then stayed constant throughout the remaining mask iterations. The increase in the *F*_1_-score in Figure 3A corresponds to the exact pattern in Figure 3D, suggesting that the performance gain was mainly from the multianswer questions; that is, the target originally intended by the masking. Multianswer questions appear to be more challenging than single-answer questions on comparing Figures 3C and 3D. According to Figures 3E and 3F, asking about multiple drugs at once made it easier for the model to find the right answer, albeit with wide performance variation. The BERT model was good at refraining from answering unanswerable questions, as indicated by the high *F*_1_-scores in Figure 3B. The detailed results of the 3 experimental runs are available in Multimedia Appendix 2. There were 189 QA entries associated with the off-label uses identified by manually reviewing 300 random drug-reason pairs from the 3 test runs, all of which happened to be single-answer cases. We computed for this small set a single aggregate *F*1-score, which was 0.49 and appeared consistently lower than the range shown in Figure 3C.

## Discussion

### Significance and Contributions

Although why-QA only covers a subdomain of clinical QA, it represents a unique category that deals with the cause, motivation, circumstance, and justification. It was estimated that 20% of the top 10 question types asked by family physicians [20] could be rephrased into a why-question. Clinical why-QA is important because (1) the ultimate task resembles expert-level explanatory synthesis of knowledge and evidence and (2) it aligns with identifying reasons for the decisions documented in clinical text. Therefore, the contents and challenges offered by the RxWhyQA data set itself have independent, practical value for developing clinical QA applications. Although drug-reason QA appears to be a niche topic, a working solution developed on the data set can broadly benefit research around adherence to clinical guidelines, care quality assessment, and health disparity from prescribing variations.

The generated RxWhyQA data set can serve as the training and testing of AI systems that target excerpting pertinent information in a clinical document to answer patient-specific questions. In addition to the unanswerable questions that require a system to refrain from extracting FP answers, the RxWhyQA data set features 9288 questions that require the system to identify multiple answers, which is a realistic challenge in clinical QA. The data set also contains 611 questions that ask about the reason for prescribing multiple drugs at once. The multianswer and multifocus questions represent a key improvement beyond existing clinical EQA data sets, of which the rigid constructs would preclude AI solutions from learning to deal with more realistic use scenarios. Additionally, our experiments on these special constructs validated the challenging nature of multianswer questions and revealed that multifocus questions may turn out to be easier due to the availability of richer information for use by the model. Our drug-reason–focused data set may offer a coherent theme that enables better controlled experiments to compare how the different QA constructs (eg, single- vs multianswer questions) affect AI system performance.

### Properties Found About the RxWhyQA Data Set

The frequent drugs and drug-reason pairs likely imply the clinical practice in the original n2c2 cohort. The finding that the top 5 drugs in the unanswerable questions (ie, no answer provided in the gold-standard annotation) were different from those in the answerable questions suggests that the prescription of certain drugs might be self-evident without needing a documented reason. Our question-answer–mentioning distance analysis showed that 90% of the drug-reason pairs were within the same or an adjacent sentence in the RxWhyQA data set, indicating modest demand for long-distance inference by AI solutions. We were able to identify frequent contextual patterns such as “PRN” (ie, pro re nata) or “as needed for” (Table 4) that AI models may learn to facilitate locating the answers. It is estimated that the data set contains 6.7% of off-label drug uses as the target answers, which would be useful for training systems to identify such cases and facilitate research on understanding the medical practice variation or innovation.

### Behavior of the Baseline EQA Solution

The notable increase in the *F*_1_-score (Figure 3D) after applying 1 round of masking suggests that the masking effectively forced the BERT model to look elsewhere, which resulted in an increase in the *F*_1_-score by retrieving the majority of the additional answers (see Table 2). Interestingly, we noticed in many cases that the model clung on to the masked span (ie, capturing the “________” as an answer) where some of such strong contextual patterns were present. This phenomenon supports that transformer-based EQA models do leverage contextual information than merely memorizing the surface question-answer pairs. Moreover, our post hoc inspection noted that correct (synonymous) answers were found by the model that were not in the gold-standard annotation (eg, “allergic reaction” versus “anaphylaxis” to a question about “epipen”), suggesting that the performance could be underestimated. As a caveat, we were aware that our baseline solution was essentially a convenient hack that made a model trained for single-answer EQA to find multiple answers through a stepwise probing procedure. As more advanced approaches constantly emerge [21,22], we welcome the research community to evaluate them by using the RxWhyQA data set. For example, the lower *F*_1_-score on those off-label uses indicates that they might represent challenging cases and demand more robust AI solutions.

### Limitations

We admit several limitations in this study: (1) the source n2c2 corpus represented a specific cohort that may not generalize to every clinical data set, (2) we did not exhaustively diversify the paraphrastic questions but left it for future exploration on other promising approaches [23], (3) we did not intend to extensively compare state-of-the-art solutions for multianswer QA but rather intended to offer a convenience baseline along with releasing the RxWhyQA corpus, (4) the drug-reason relations represent a narrow topic for EQA development and evaluation. However, we believe that the definite theme would preferably make it a less confounded test set for assessing the effect of multianswer and multifocus questions on AI systems.

### Conclusions

We derived and shared the RxWhyQA, an EQA data set for training and testing systems to answer patient-specific questions based on clinical documents. The RxWhyQA data set includes 9288 multianswer questions and 611 multifocus questions, each representing a critical scenario not well covered by existing data sets. Upon evaluating a baseline solution, the multianswer questions appeared to be more challenging than single-answer questions. Although the RxWhyQA focuses on why-questions derived from drug-reason relations, it offers a rich data set involving realistic constructs and exemplifies an innovation in recasting NLP annotations of different tasks for EQA research.

## Appendix

Multimedia Appendix 1 Manual annotation of off-label uses in 300 randomly sampled drug-reason QA pairs from the test sets.

Multimedia Appendix 2 Detailed *F*_1_-scores of the BERT model across three test runs, on the different subsets, with applying the incremental answer-masking.

## Abbreviations

AI: artificial intelligence
BERT: Bidirectional Encoder Representations from Transformers
dev: develop
EHR: electronic health record
EQA: extractive question-answering
FN: false negative
FP: false positive
n2c2: National NLP Clinical Challenges
NLP: natural language processing
QA: question-answering
SQuAD: Stanford Question Answering Dataset
TP: true positive
